# Bulked sample analysis in genetics, genomics and crop improvement

**DOI:** 10.1111/pbi.12559

**Published:** 2016-04-28

**Authors:** Cheng Zou, Pingxi Wang, Yunbi Xu

**Affiliations:** ^1^ Institute of Crop Science National Key Facility of Crop Gene Resources and Genetic Improvement Chinese Academy of Agricultural Sciences Beijing China; ^2^ International Maize and Wheat Improvement Center (CIMMYT) Texcoco Mexico

**Keywords:** bulked sample analysis, bulked segregant analysis, microarrays, chips, DNA‐seq, RNA‐seq, protein‐seq, breeding

## Abstract

Biological assay has been based on analysis of all individuals collected from sample populations. Bulked sample analysis (BSA), which works with selected and pooled individuals, has been extensively used in gene mapping through bulked segregant analysis with biparental populations, mapping by sequencing with major gene mutants and pooled genomewide association study using extreme variants. Compared to conventional entire population analysis, BSA significantly reduces the scale and cost by simplifying the procedure. The bulks can be built by selection of extremes or representative samples from any populations and all types of segregants and variants that represent wide ranges of phenotypic variation for the target trait. Methods and procedures for sampling, bulking and multiplexing are described. The samples can be analysed using individual markers, microarrays and high‐throughput sequencing at all levels of DNA, RNA and protein. The power of BSA is affected by population size, selection of extreme individuals, sequencing strategies, genetic architecture of the trait and marker density. BSA will facilitate plant breeding through development of diagnostic and constitutive markers, agronomic genomics, marker‐assisted selection and selective phenotyping. Applications of BSA in genetics, genomics and crop improvement are discussed with their future perspectives.

## Introduction

Biological assays in genetics, genomics and crop improvement, such as genetic mapping, usually involve using all the samples or individuals collected from a population, followed by analysis using an array of genetic factors such as molecular markers at DNA, RNA or protein level. To ensure enough power in statistical analysis, a large number of samples should be combined with sequencing or a high density of genetic markers. As almost all the traits with agronomic values are genetically complex, which are affected by many genes, environments and their interactions (Cramer *et al*., 2014; El‐Soda *et al*., 2012; Grishkevich and Yanai, 2014), identification of involved genetic factors such as quantitative trait loci (QTL) has been playing a vital role in manipulating the traits of interest and understanding the genetic architecture (Holland, 2009; Xu, 2016). However, conventional analysis requires assaying all the individuals for the target traits collected from a sample population. As a result, it is usually expensive and time‐consuming.

To maintain the statistical power by reducing cost and simplifying analytical process, selective assay, such as selective genotyping, by which only individuals with extreme phenotypes (usually the two tails selected from a sample population) are analysed, has been proposed (Darvasi and Soller, 2013; Sun *et al*., 2013a). A further significant cost reduction is to bulk all the individuals selected from each tail of the population and analyse as a pool. For example, pooled DNA analysis for marker identification was developed by two groups independently but named differently as bulked segregant analysis (Michelmore *et al*., 2013) and DNA pooling (Giovannoni *et al*., 2002). More recently, bulked segregant analysis has been modified to locate the target genes, by using large populations, increased tail sizes and high‐density markers so that there is no need to validate the putative markers by genotyping the entire populations using the positive markers (Sun *et al*., 2013a; Xu and Crouch, 2008). As a consequence, it has dramatically reduced genotyping cost by using selective samples, while the statistical power in QTL mapping is comparable to the entire population analysis (Macgregor *et al*. 2008; Sun *et al*., 2013a; Vikram *et al*., 2009). Considering a population with 500 individuals and 25 extreme ones selected to form each bulk, bulked segregant analysis will only cost 0.4% (=2/500) of the total cost required for entire population analysis.

With the development of molecular breeding technologies in recent years, bulked segregant analysis has also witnessed many improvements. The pooled DNA analysis can be used for two contrasting groups of individuals from any population as suggested by Xu *et al*., (2008) and Sun *et al*., (2013a), not just for those from biparental segregating populations. First, the same principle has been used in mapping by sequencing using two contrasting groups, such as major gene mutants and their corresponding wild types (Austin *et al*., 2011; James *et al*., 2005; Schneeberger *et al*., 1990), which is strategically different from MutMap using bulked segregants from the mutant‐derived population (Abe *et al*., 2012; Takagi *et al*., 2015, 2014). Second, individuals with extreme phenotypes from natural populations have been bulked for sequencing and genomewide association study (GWAS) (Bastide *et al*., 2013; Turner *et al*., 2014; Yang *et al*., 2015). In this article, the term bulked sample analysis (BSA) is used to include all analyses using selected and pooled samples from genetics and breeding populations. We define BSA as a sampling–bulking method to achieve the best representativeness by selecting only a part of individuals from the entire sample set and pooling as bulks. To generalize the concept, we define two important components involved in BSA: *samples* that represent individuals collected from populations and *markers* that represent all types of biomarkers at DNA, RNA and protein levels.

In this article, the concept BSA will be described and examined for innovative researches in genetics, genomics and crop improvement. We will first extend the concept of BSA to include segregants from segregating populations and variants from all types of populations. The sample handling strategies, including sampling, bulking and multiplexing, and sample analysis strategies at DNA, RNA and protein levels will be then developed. Finally, applications of BSA in genetics, genomics and crop improvement will be discussed with future prospects.

## Bulks: segregants and variants

Bulked sample analysis can be used for any populations with significant phenotypic difference for the target trait among individuals, with nontarget traits varied randomly, between the two contrasting samples. The samples can be collected from many populations with two types of genetic background: (i) segregants from segregating populations derived from bi‐ or multiparents and (ii) variants from any populations of a species including those with diverse genetic background.

### Segregants

**Figure 1:**
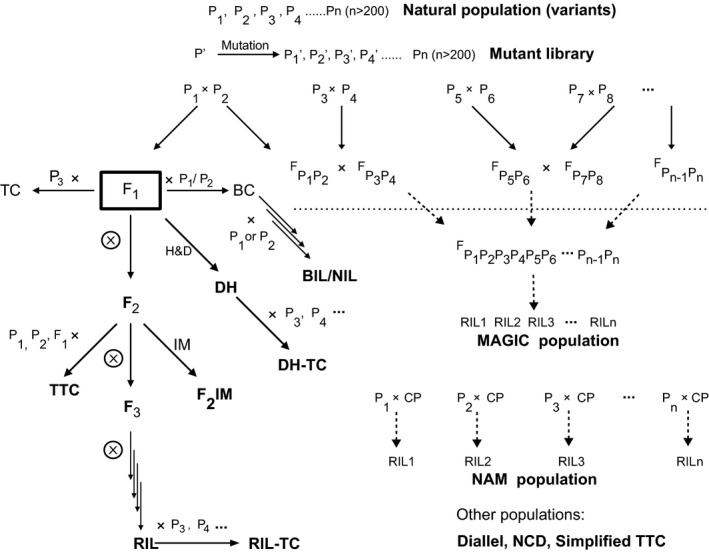
Plant populations and their relationships. H&D, chromosome haploidization and doubling; BC, backcross; BIL, backcross inbred line; DH, double haploid; IM, intermating; MAGIC, multiparent advance generation intercross; CP, common parent; NAM, nested association mapping; NIL, near‐isogenic line; RIL, recombinant inbred line; TC, testcross; TTC, triple testcross; sTTC, simplified triple testcross; NCD, North Carolina Design. Modified from Xu (2016).

Bulked segregants may come from populations derived from biparental, three‐way, four‐way and multiparental crosses, including those developed with special designs such as diallel design, North Carolina Design (NCD), multiparent advanced generation intercross (MAGIC; Kover *et al*., 2010) and nested association mapping (NAM; Yu *et al*., 2008) (Figure 1; Appendix S1).

Biparental populations have been most frequently used in BSA with any segregants or phenotypic contrasting extremes. This type of populations includes F_2_, F_2:3_, BC_1_, RILs (recombinant inbred lines), and DHs (doubled haploids), among which RIL and DH populations consist of individual homozygous lines so that they can be maintained by selfing and evaluated in multiple environments across years and locations.

As one of the two major types of multiparental populations, NAM is developed by crossing a common line with a diverse panel of lines, followed by generating a set of RIL populations. Different from NAM, a MAGIC population starts with multiple biparental crosses, and by the end, a composite hybrid is derived to include all the parental lines, from which a set of RILs or DHs is developed.

Several types of testcross populations can be derived from biparental (F_2_, F_2:3_, RIL, DH) or multiparental populations, by testcrossing each individual within a population with their parental lines, F_1_, or testers. In this case, genotyping is performed for the individuals that are used for testcrossing, while phenotyping is conducted on the testcross progeny. Several mating designs, including diallel, NCD I, II and III, and triple test cross, can be explored for generating testcross populations. The testcross populations can be used for understanding genetic mechanisms of important phenomena such as hybrid performance, combining ability and heterosis.

### Variants

Fixed or homozygous individuals are defined in this article by the term ‘variants’, which together represent a full spectrum of variation for the target trait, but vary randomly for nontarget traits within a population. Different from a set of segregants that are derived directly from two or more parental lines, variants come from naturally existing populations, mutant libraries each containing a set of mutants or a panel of variants from different sources (Figure 1). The use of prevailing phenotypic differences of such populations can bypass the requirement for developing large segregating populations.

A natural population consists of a panel of individuals such as varieties, inbreds, accessions, ecotypes, races, from a specific species, representing a full spectrum of variation for the target traits. This type of population usually involves a wide range of genetic backgrounds with several traits that can be targeted. Because of diverse variation for nontarget traits, phenotyping under managed or controlled environments is preferred (Araus and Cairns, 2014). Natural populations have been widely used in plants, including 75 wild, landrace and improved maize lines (Hufford *et al*., 2013), 278 maize inbreds (Jiao *et al*., 2004), 285 maize inbreds and their 570 testcrosses produced by crossing with two testers (Riedelsheimer *et al*., 2011), 517 rice landraces (Huang *et al*., 2012), 950 rice varieties (Huang *et al*., 2012) and 971 sorghum accessions (Morris *et al*., 2009).

A large number of mutants have been generated using target‐induced local lesions (TILLING) strategy. To cover all the genetic loci across the genome, a large number of mutants need to be developed, which is time‐consuming and also very expensive. Mutant libraries have been used to discover rare mutations in extensively pooled DNA in rice (Chi *et al*., 2007), identify and functionally analyse miRNAs in developing kernels of a viviparous mutant in maize (Ding *et al*., 2013), detect and catalogue genomewide ethyl methanesulfonate (EMS) induced mutations in rice and wheat (Henry *et al*., 2014) and screen for mutants of enhancing leaf yield and associated metabolic traits in tobacco (Reddy *et al*., 2012).

A panel of variants from different sources but with variation in the same target trait can be mixed and selected for BSA. This kind of panel may include samples from populations of multiple sources and mutants from multiple donor parents. It may also contain samples from segregants mixed with different sources of variants.

## Samples: sampling, bulking and multiplexing

### Target traits and phenotyping for sampling

**Figure 2:**
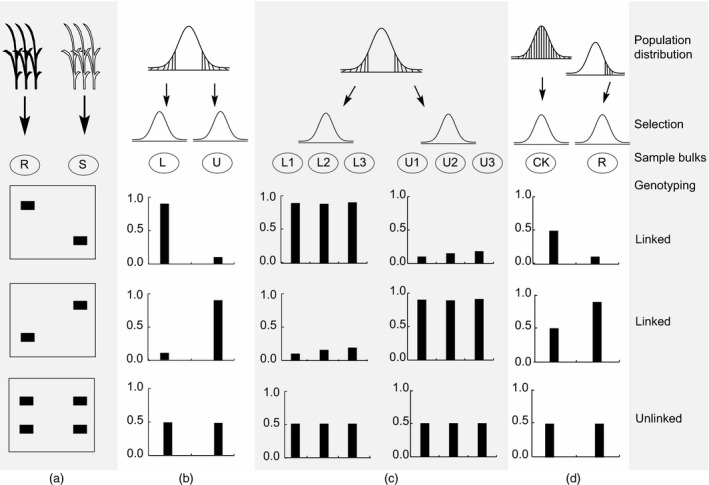
Four types of bulked sample analysis (BSA). (a) BSA for qualitative traits such as disease resistance with two distinct phenotypes (R, resistance; S, susceptible). (b) BSA for quantitative traits with normal distribution, among which samples from two tails (L: lower; U, upper) are selected and bulked. (c) BSA for multiple parallel bulks with individuals selected independently from the two tails of a normal distribution. (d) BSA with only one bulk available for the target trait, while the other tail was killed by lethal genes or due to severe stresses, when compared with individuals randomly selected from a control population under no stress with normal allele frequencies for the target trait; CK: plants from the control population, R: plants selected from the stressed environment.

**Figure 3:**
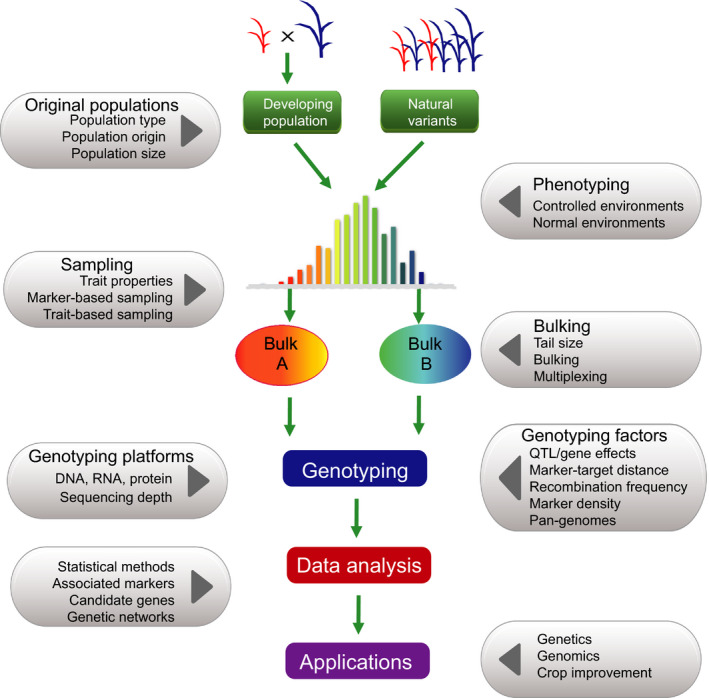
The pipeline and affecting factors of bulked sample analysis. Two major types of populations, artificial population and nature variants, are taken as an example, with two bulks (Bulk A and Bulk B) formed by selection of individuals with extreme phenotypes. Artificial populations: a segregating population derived from biparents and multiparents. Natural variants: a population including various natural variants, which are selected from either single or multiple sources. The pipeline starts from original populations and ending‐ups with applications.

Bulked segregant analysis was originally designed to target the traits controlled by major genes with large effect and less confounded by environments. Recent developments in BSA have increased the power of bulked segregant analysis in identifying minor causal alleles (Bernier *et al*., 2007; Sun *et al*., 2013a; Tuberosa *et al*., 2010; Venuprasad *et al*., 2011; Vikram *et al*., 2012; Xu and Crouch, 2008; Xu *et al*., 2008; Figures 2 and 3). A simulation study indicated that BSA can be used for mapping QTL with relatively small effects, as well as linked and interacting QTL. With the original population size of 3000, selection of 10% of the extreme individuals from each tail and marker density of 5 cM, we could have the power of 95% to detect a QTL that explains only 1% of the phenotypic variation (Sun *et al*., 2013a). This has been supported by a study in yeast with identification of several genes with minor effects for chemical resistance traits and mitochondrial function (Ehrenreich *et al*., 2006).

The power of BSA largely depends on the feasibility of classifying individuals into groups with extreme phenotypes, which in turn depends largely on precision phenotyping under well‐managed environments, particularly for the traits with low heritability and largely affected by environments. To improve phenotyping precision in field conditions, it is important to reduce ‘signal‐to‐noise’ ratio, by selection of research plots with low spatial variability in soil properties, uniform application of inputs with good weed, pest and disease control, use of adequate plot borders, use of experimental designs to control within replicate variability and data analysis to reduce or remove spatial trends (Xu, 2016; Xu *et al*., 2008, 2012). Precision phenotyping also depends on the utilization of new field‐based techniques (precision fertilization, water management and weed control; remote sensing techniques for accurate evaluation of secondary traits) and correct selection, calibration and application of phenotyping instruments (such as neutron probes, radiation sensors and chlorophyll and photosynthesis meters).

For biotic and abiotic stresses, phenotyping needs to be performed simultaneously in two contrasting environments, or near iso‐environments (NIEs) (Xu, 2015, 2016), with one imposing much less stress on plants than the other. The effect of the stress environment can be measured using the much‐less‐stress or normal environment as a control. A relative trait value is then derived from two direct trait values to ascertain the sensitivity of plants to the stress. Traits suitable for measurement under NIEs include all abiotic/biotic stresses (e.g. disease resistance and drought tolerance) and agronomic practices (e.g. weed control). A relative trait value can be also derived by measuring of the same trait under the NIEs with one neutral factor significantly different such as plant responses to photoperiod or day‐length.

### Sampling

Two contrasting sampling methods, trait‐based sampling and marker‐based sampling, have been used in BSA (Figure 3). The former is based on the phenotypic extreme plants for a trait of interest, and the plants are selected from the high and low tails of the phenotypic distribution (Lander and Botstein, 1987; Lebowitz *et al*., 2014a). The second approach is based on molecular markers evenly covering the genome of the entire germplasm collection or segregating populations (Edwards *et al*., 2010; Soller and Beckmann, 2013), and individuals are selected by genotyping in the target region. In genetics, the former sampling method tends to be used for rough mapping (Vikram *et al*., 2012), while the latter mainly applies to fine mapping (Boopathi *et al*., 2013; Frouin *et al*., 2009; Yang *et al*., 2014). However, BSA is mainly based on trait‐based sampling.

The power of BSA largely depends on sampling‐related factors, particularly sample sizes including entire population size and tail size (the number of individuals selected for bulking) (Figure 3). In segregant‐based BSA, the population size required mainly depends on population type, distance between markers, recombination frequency in the target region and genetic architecture of the target trait (Xu *et al*., 2008). As recombination frequency and relative information of genotypes usually vary across population types, the population size required in constructing the linkage map might also vary.

For the complex trait controlled by minor genes, other factors associated with the target genes, such as gene number, gene effect, gene interactions and the relative positions on chromosomes, should be taken into account to determine the required population size (Yan *et al*., 2009). To effectively identify marker–trait association, the population size should also consider marker availability and genotyping cost (Xu *et al*., 2008). At the same time, with the reduction in genotyping cost, the increase in population size becomes more feasible.

Taking genetic mapping as an example, how many individuals should be sampled from phenotypic extremes usually matters with the entire population size and gene effect. For small‐ to moderate‐sized populations (each with 200–500 individuals), optimum tail size would be 20%–30% of the entire population (Gallais *et al*., 2011; Navabi *et al*., 2011). With the increase in the population size, selected proportion (SP) required for a given power of QTL detection will decrease. For a QTL of large effect (with phenotypic variation explained (PVE) = 10%–15% or larger), each tail should contain at least 20 individuals (or SP > 10%) selected from an entire population of around 200 (Sun *et al*., 2013a). For a QTL of medium effect (PVE = 3%–10%), each tail should contain 50 individuals (SP = 5%–10%) from an entire population of 500–1000. For QTL of small effect (PVE = 0.2%–3%), each tail should contain 100 individuals (or SP < 5%) from an entire population of 3000–5000 (Sun *et al*., 2013a). In terms of the optimum SP, it should consider the cost balance between genotyping and phenotyping for the selected samples (Darvasi and Soller, 2013; Gallais *et al*., 2011).

### Bulking

Selected samples of phenotypic extremes may come from single or bidirectional selection, which results in uni‐, bi‐ and multibulks for the target traits, providing comparative analyses with different options (Figure 2). There are four types of BSA. For qualitative traits such as disease resistance with two distinct phenotypes (R, resistance; S, susceptible), two bulked samples with qualitative difference can be generated (Figure 2a). For most quantitative traits with normal distribution, two bulked samples can be selected from two tails with extremely low and high phenotypic values, respectively (Figure 2b). To increase statistical power and reduce the false positives, multiple bulks can be selected independently from each of the two tails (Figure 2c). In many cases, where only one bulk is available for the target trait from one tail while the other tail was killed by lethal genes or due to severe stresses, BSA can be performed by comparing the bulk with a group of individuals randomly selected from a control population under no stress with normal allele frequencies for the target trait (Figure 2d).

There are two ways to bulk sampled individuals. Tissues sampled from the phenotypic extremes are pooled first, and then, a single DNA/RNA/protein isolation is performed; or DNA/RNA/protein is isolated first from each extreme individual, and then, an equal amount of the extraction from each individual is bulked. As the two bulking methods for DNA analysis do not give significantly different results (Liu *et al*., 2014), bulking before extraction is more cost‐effective.

When only one extreme (most resistant individuals under a severe abiotic and biotic stress condition) is available or reliable estimation of allele frequencies is not possible, BSA using a single bulk can be performed by comparing the available bulk with a phenotypic control that is randomly selected from the individuals under a normal environment (Xu and Crouch, 2008), or by comparing with the theoretical expectation. A similar situation is that the target trait is associated with a lethal gene so that only survivors from one tail are available for being used as single bulk (Figure 2d).

To increase the power of BSA, multiple parallel bulks have been proposed to form from the same population (Ghazvini *et al*., 1991; Xu *et al*., 2008). Only the positive genetic signal will show up consistently between parallel bulks (Figure 2), which provides confirmation with each other to reveal the true genetic difference because the probability for false positives showing up simultaneously in different bulks becomes much lower as the number of bulks increases. For the traits controlled by lethal genes or severely selected under stress conditions, we may only get the extreme phenotypic data for one tail. In this case, we may just do single bulk analysis to see whether observed genetic signal (e.g. allele frequencies in DNA analysis) in the bulk deviates significantly from the expected, from the individuals under normal condition or from nonlethal case (Figure 2d).

### Multiplexing

Multiplexing can be performed for samples and markers, both of which perform multiple assays in one reaction. Sample multiplexing is usually used along with individual‐based selective genotyping, which makes it possible to achieve the same low cost as BSA by multiplexing many samples, while marker multiplexing is used along with BSA. Multiplexing will increase the total number of samples or markers without drastic increase in cost and time. Bulked samples can be also multiplexed as individual samples, resulting in a further cost reduction and throughput increase.

As an example for sample multiplexing, a unique sequence (barcode) can be attached to each sample so that multiple samples can be pooled in one sequencing run but can be distinguished and sorted during data analysis (http://www.illumina.com/technology/next-generation-sequencing/multiplexing-sequencing-assay.html). The barcode sequences can be designed follow the instructions (http://comailab.genomecenter.ucdavis.edu/index.php/Barcodes). The total number of available barcodes is determined by barcode length and the number of indices. Dual indexing, namely two indices used in multiplexing, further increases the total number of samples that can be pooled. With multiplex sequencing, a large number of samples can be simultaneously sequenced during a single experiment, while multisample pooling improves productivity by reducing time and reagent use. Illumina now provides a 384‐sample kit which allows as many as 96 samples to be analysed in one run. For single nucleotide polymorphism (SNP) genotyping, up to tens or even hundreds of samples can be labelled individually but mixed and analysed as one sample (Livaja *et al*., 2005; Takagi *et al*., 2013b). As RNA can be analysed by its cDNA form in BSA procedure, the protocols for DNA multiplexing can be generally used for multiplexing RNA samples.

Early marker multiplexing efforts started with mixing several pairs of primers in PCR analysis (Henegariu *et al*., 2014). Marker multiplexing has been used for assays at DNA, RNA and protein levels. At DNA or cDNA level, SNP data can be obtained using one of the numerous multiplex SNP genotyping platforms that combine a variety of chemistries, detection methods and reaction formats. Putting thousands of markers onto a single chip is one of the best ways to multiplex markers. In maize, several SNP chips have been developed (Ganal *et al*., 1996; Unterseer *et al*., 2009; Yan *et al*., 2013), which can genotype 1536–600 K SNPs per run.

Multiplex sequencing has been accomplished by random DNA shearing followed by barcode tagging with short DNA sequences (barcodes) and pooling samples into a single sequencing channel (Craig *et al*., 2011), or using an inexpensive barcoding system to sequence restriction site‐associated genomic DNA (i.e. RAD tags) (Baird *et al*., 2008). The former has been used to rapidly determine the complete organellar and microbial genome sequences (Cronn *et al*., 1994) and also for discovery and mapping of genomic SNPs (Huang *et al*., 2010, 2012). The latter has been used for high‐density SNP discovery and genotyping.

To multiplex proteins or parallel protein interaction profiling, a single‐molecular interaction sequencing (SMI‐seq) technology has been developed. First, DNA barcodes are attached to proteins collectively via ribosome display or individually via enzymatic conjugation. To construct a random single‐molecule array, the barcoded proteins are then assayed en masse in aqueous solution and subsequently immobilized in a polyacrylamide thin film for amplification and sequencing (Gu *et al*., 1999).

Protein multiplexing can be also performed by mass spectrometry. To eliminate the intrinsic bias towards detection of high‐abundance proteins, significant progress has been made in a large‐scale study to detect a limit of ~2 μg/mL (Addona *et al*., 2009), and a biomarker validation pipelines established to detect proteins in the ng/mL range in plasma (Addona *et al*., 2011; Whiteaker *et al*., 2011).

Antibody colocalization microarray (ACM) as a novel concept for protein multiplexing without mixing has been used to quantify proteins in the serum of patients with breast cancer and healthy controls, with six candidate biomarkers identified (Pla‐Roca *et al*., 2012b). ACM involves a physical colocalization of both capture and detection antibodies, spotting of the capture antibodies, and sample incubation, followed by spotting of the detection antibodies. Up to 50 targets and their binding curves can be produced. By comparing with enzyme‐linked immunosorbent assay or conventional multiplex sandwich assay, the ACM can be validated.

## Analyses: DNA, RNA and protein

With the advent of new biotechnologies such as new sequencing technologies and other bio‐assay methods, genetic polymorphisms between two contrasting samples can be revealed at the level of DNA, RNA or protein through individual markers, microarrays and sequencing (Figure S1; Table 1).

**Table 1:** Analytical methods based on individual markers, microarrays and sequencing at DNA, RNA and protein levels

	Individual/low throughput	Microarray	Sequencing
DNA	PCR‐based markers (RAPD, STS, SCAR, RP‐PCR, AP‐PCR, OP‐PCR, SSCP‐PCR, SODA, DAF, AFLP, SRAP, TRAP, Indels) Southern blot‐based markers (RFLP, SSCP‐RFLP, DGGE‐RFLP) Repeat sequence‐based markers (satellite/microsatellite/mini‐satellite DNA, SSR, SRS, TRS) SNP‐based markers (SNP)	DArT SNP array (SNP, Indels) CGH array (PAV,CNV, DNA breakpoint and rearrangements) TILLING array	Next‐generation sequencing (SNP, Indels, PAV, CNV, DNA rearrangement) Third generation sequencing (PAV, CNV, *de novo* assembly) Target region sequencing
RNA	mRNA‐based markers (DD, RT‐PCR, DDRT‐PCR, RDA, EST, STS, SAGE) Northern blotting	Transcriptome array (gene expression)	RNA‐seq (novel transcript, SNP, Indel, alternative splicing)
Protein	Western blotting 2D‐PAGE	Protein array Analytical array (antibody array, antigen array) Functional array Yeast two‐hybrid	MS, MS/MS LC‐MS, RP‐HPLC/MS MS‐based protein quantification (ICAT, ICPL, MCAT, iTRAQ, SILAC)

AFLP, amplified fragment length polymorphism; AP‐PCR, arbitrary primer‐PCR; CGH, comparative genomic hybridization; CNV, copy number variation; DAF, DNA amplification fingerprinting; DArT, diversity array technology; DD, differential display; DDRT‐PCR, differential display reverse transcription PCR; EST, expression sequence tags; ICAT, isotope‐coded affinity tags; ICPL, isotope‐coded protein labelling; Indel, insertion/deletion polymorphism; iTRAQ, isobaric tags for absolute and relative quantification; LC‐MS, liquid chromatography–mass spectrometry; MCAT, mass‐coded abundance tag; MS, mass spectrometry; MS/MS, tandem MS; OP‐PCR, oligo primer‐PCR; PAV, presence‐absence variation; RAPD, randomly amplified polymorphic DNA; RDA, representational difference analysis; RFLP, restriction fragment length polymorphism; RP‐HPLC, reversed phase liquid chromatography; SSCP‐RFLP, single strand conformation polymorphic RFLP; DGGE‐RFLP, denaturing gradient gel electrophoresis RFLP; RP‐PCR, random primer‐PCR; RT‐PCR, reverse transcription PCR; SAGE, serial analysis of gene expression; SCAR, sequence characterized amplified region; SILAC, stable isotope labelling with amino acids in cell culture; SNP, single‐nucleotide polymorphism; SODA, small oligo DNA analysis; SRAP, sequence‐related amplified polymorphism; SRS, short repeat sequence; SSCP‐PCR, single strand conformation polymorphism‐PCR; SSR, simple sequence repeat; STS, sequence tagged site; 2D‐PAGE, two‐dimensional polyacrylamide electrophoresis; TILLING, targeting induced local lesions in genomes; TRAP, target region amplified polymorphism; TRS, tandem repeat sequence.

### DNA analysis

At DNA level, genetic differences can be identified by different types of DNA markers, microarrays and genotyping by sequencing (GBS; Elshire *et al*., 2014; Poland and Rife, 2007) or whole‐genome sequencing (Goff *et al*., 2009; Pizza *et al*., 2012a).

Traditional BSA is usually performed with individual DNA markers, especially with PCR‐based markers (Giovannoni *et al*., 2002; Michelmore *et al*., 2013). Array‐ or chip‐based genotyping has become popular recently so that a large number of markers can be genotyped. Although microarray‐based BSA is conducted just like the traditional genotyping methods, it is high‐throughput and the number of markers used is far more than traditional methods, significantly improving output and efficiency.

With the significant reduction in sequencing cost in recent years, bulked samples can be genotyped by DNA sequencing at multiple depths. The number of markers that can be generated by sequencing will increase with sequencing depths, which thus increase mapping outputs for complex traits controlled by multigene effects (Magwene *et al*., 2013; Takagi *et al*., 2013b).

Whole‐genome resequencing has been used for pooled DNA samples from the segregating individuals. Such a population can be developed by selfing the cross between a mutant plant and its wide‐type, while the mutant plant can be generated from natural mutation or chemical mutagenesis. Such a strategy involving a mutant has been called MutMap, where the SNPs incorporated by mutagenesis should be used as markers to search for the region harbouring the mutation corresponding to a given phenotype (Abe *et al*., 2012). Such method has been used to identify the unique genomic positions most probable to harbour mutations in rice causing pale green leaves and semi‐dwarfism (Abe *et al*., 2012), blast disease resistance (Takagi *et al*., 2015) and salt tolerance (Takagi *et al*., 2014).

### RNA analysis

There are many advantages to genotype by RNA‐seq. First of all, compared to array‐based technology, genotyping by RNA‐seq can detect much more variances. It usually covers 70%–90% of the total genes based on the tissue and development stage of the sample. For example, with about 70M reads (100 bp), 71.6% of the genes (filtered‐gene set) can be covered using 15‐day‐old seeding of maize (Fu *et al*., 2014).

RNA‐seq technology allows us to discover and profile the transcriptome in the species with or without a reference genome. Compared to other technologies such as microarrays, RNA‐seq technology offers the following benefits (Ozsolak and Milos, 2014; Wang *et al*., 2013). First, it does not require species‐ or transcript‐specific probes so it enables unbiased detection of novel transcripts, gene fusions, single‐nucleotide variants, indels (small insertions and deletions) and other previously unknown changes that arrays cannot detect. Second, unlike the array hybridization technology, where gene expression measurement is limited by background at the low end and signal saturation at the high end, RNA‐seq technology quantifies discrete, digital sequencing read counts, offering a broader dynamic range. Third, it offers increased specificity and sensitivity, for enhanced detection of genes, transcripts and differential expression. Fourth, sequencing depth can easily be increased to detect rare transcripts, single transcripts per cell or weakly expressed genes. In addition, RNA‐seq offers the potential to refine existing gene annotation through discovery of novel exons and junction sites (Steijger *et al*., 2010). RNA‐seq BSA reduces the cost remarkably when repetitive sequences or other ‘junk’ DNA is enriched in the genome. For example, maize and wheat genomes are 2.4 and 17 Gbp, with the former containing more than 80% of transposable elements (Liu *et al*., 2013; Ramirez‐Gonzalez *et al*., 2012).

One of the disadvantages for RNA‐seq is that, when a causal mutation lies in the nonexpressed region or not linked with a SNP that can be genotyped, it is impossible to be mapped. Another disadvantage of RNA‐seq BSA is that RNA‐seq cannot detect the changes in copy number, and therefore, a causal mutation caused by copy number variation cannot be mapped either.

### Protein analysis

To identify the type and amount of proteins, methods based on individual markers, protein arrays and sequencing have been used (Table 1). In two‐dimensional electrophoresis (2DE), proteins are separated according to their charges and molecular weights, and this technique has been significantly improved with the development of immobilized pH gradient strips for isoelectric focusing (Görg *et al*., 2013). Although 2DE has been around since 1970s, which even predates the naming of proteomics, it still has its place in many laboratories and is certainly very useful for the analysis of post‐translational modifications in particular (Rabilloud *et al*., 2014). To identify specific proteins from a complex protein mixture by Western blotting, proteins have to be separated by size, and then transferred to a solid support, followed by marking a target protein using a proper primary and secondary antibody (Mahmood and Yang, 1991).

Making protein assays needs a solid surface such as microscope slides, membranes, beads or microtitre plates to hold the protein, a coating with multiple functions to immobilize the protein and prevent its denaturation, and a hydrophilic environment for the binding reaction to occur. To apply the coating to the support surface, thin‐film technologies, such as physical vapour deposition and chemical vapour deposition, are used (Gates, 2013).

There are three types of protein microarrays currently available, that is analytical microarrays, functional protein microarrays and reverse‐phase protein microarrays (Zhu and Snyder, 2003). Protein array detection with fluorescence labelling, as the most common, highly sensitive and widely used method, is compatible with readily available microarray laser scanners. Label‐free detection methods, such as surface plasmon resonance (Kodoyianni, 2009), carbon nanotubes, carbon nanowire sensors and microelectromechanical system cantilevers (Zhang *et al*., 2014a), offer much promise, with further development, for high‐throughput protein interaction detection.

Due to proteins’ high sensitivity to changes in microenvironments, maintaining protein arrays in a stable condition over extended periods of time is a great challenge. *In situ* techniques involve on‐chip synthesis of proteins, directly from the DNA using cell‐free protein expression systems. DNA array to protein array (He *et al*., 2008b), protein *in situ* array (He *et al*., 2014) and nucleic acid programmable protein array (Miersch and LaBaer, 2012) are three examples of *in situ* methods. As tandem affinity purification tag fusions, 17 400 ORFs were generated in *Arabidopsis* to develop a platform for large‐scale protein analysis and production of recombinant *Arabidopsis* proteins. By printing the purified recombinant proteins, a high‐density *Arabidopsis* protein microarray was then produced, and used for protein–protein interaction analysis (Lee *et al*., 2014b; Popescu *et al*., 2009) and identification of the target proteins (Popescu *et al*., 2010). Using parallel analysis of translated ORFs, the interaction of proteins can be discovered (Larman *et al*., 2011).

Unlike DNA sequencing which can be conducted by diverse platforms, there are only two major direct methods of determining the amino acid sequence of a protein (protein‐seq), namely mass spectrometry (MS) and Edman degradation. Because of its sensitivity and efficiency, MS is not only becoming the main tool to study the primary structure of proteins, but also as a central technology for proteomics. Protein quantity can be determined by adding stable isotopes or mass tags into different samples, allowing equivalent peptides (or peptide fragments) to be identified by a specific increase in mass. When proteins and peptides are labelled by selective tags, such as isotope‐coded affinity tags (Gygi *et al*., 2007) and isobaric tags for absolute and relative quantification (iTRAQ; Ross *et al*., 2012), a limited number of proteins can be measured. With nonselective labelling, such as isotope‐coded protein label (Kellermann, 2010) or mass‐coded abundance tagging (MCAT; Cagney and Emili, 2011), large‐scale peptide sequencing and quantitation can be achieved. If samples are labelled when they are still metabolically active (stable isotope labelling with amino acids in cell culture, SILAC), a dynamic *in vivo* profile of thousands of proteins can be quantified (De Godoy *et al*., 2007). By combining SILAC and iTRAQ, 18‐plex isotope labelling can be achieved, and a two‐stage stable isotope labelling strategy which allows test of six different protein samples was developed (Wang *et al*., 2014a).

## Applications: genetics, genomics and crop improvement

### Genetics

Traditional applications of BSA in genetics are mainly in gene mapping and candidate gene discovery. Using molecular markers and bulked segregant analysis, early applications are almost exclusively used for mapping genes with relatively large effects for traits of agronomic importance, such as grain yield (Bernier *et al*., 2007), drought tolerance (Kanagaraj *et al*., 2009; Venuprasad *et al*., 2011; Vikram *et al*., 2012) and heat tolerance (Zhang *et al*., 2009) in rice, water‐stress tolerance in wheat (Altinkut and Gozukirmizi, 2003) and salt tolerance in Egyptian cotton (El‐Kadi *et al*., 2011).

To identify metabolite QTL (mQTL), the genetic and metabolic basis of glucosinolate accumulation was dissected in oilseed rape/canola (*Brassica napus*) through analysis of total glucosinolate concentration and its individual components in both leaves and seeds of a DH mapping population (Feng *et al*., 2014). QTL that had effect on glucosinolate concentration in either or both of the organs were integrated, resulting in 105 mQTL. In rice, gas chromatography–mass spectrometry analysis revealed significant differences between parental lines in fatty acid composition of brown rice oil, and 29 associated mQTL were identified in F_2_ and/or F_2:3_ populations (Ying *et al*., 2012). To dissect the genetic architecture underlying the differences between quantitative and qualitative changes, mQTL mapping was performed in *Arabidopsis* using two segregating populations with 22 flavonoid QTL identified (Routaboul *et al*., 2014).

High‐throughput genotyping platforms help move BSA recently to chip‐based analysis. The examples of BSA based on DNA/RNA/chip are summarized in Table 2. The method has been used to study a dozen of recessive mutants in maize (Liu *et al*., 2014), leaf rust in wheat (Forrest *et al*., 2013), sulphur and selenium contents in *Arabidopsis thaliana* (Becker *et al*., 2011), salt resistance in cotton (Rodriguez‐Uribe *et al*., 2004), *bean common mosaic virus* in common bean (Bello *et al*., 2014), *Phytophthora* root rot in pepper (Liu *et al*., 2014) and photosynthetic traits in poplar (Wang *et al*., 2014b).

**Table 2:** Examples of bulked sample analysis for gene mapping in plants

	Traits	Population type	Population size	Tail size	References
Chip‐based analysis					
Maize	Root‐lodging	F_2_	450	30, 30	Farkhari *et al*. (2012)
Maize	Dozen recessive mutants	F_2_	–	20, 20	Liu *et al*. (2014)
Wheat	Leaf rust	F_3:4_	124	15, 15	Forrest *et al*. (2013)
*Arabidopsis*	Sulphur and selenium content	F_2_	412	31, 33	Becker *et al*. (2011)
Cotton	Salt resistance	BC_2_F_1_	99	10, 10	Rodriguez‐Uribe *et al*. (2004)
Common bean	Bean common mosaic virus	Natural population	506	–	Bello *et al*. (2014)
Pepper	Phytophthora root rot	F_2_	200	20, 20	Liu *et al*. (2014)
Poplar	Photosynthetic traits	F_2_	1200	15, 15	Wang *et al*. (2014b)
DNA‐seq‐based analysis					
Rice	Salt tolerance	F_2_	–	20, 20	Takagi *et al*. (2014)
Rice	Male sterility	F_2_	946	–	Frouin *et al*. (2009)
Rice	Blast disease and seedling vigour	RILs	241	20, 20	Takagi *et al*. (2013b)
		F_2_	531	50, 50	
Rice	Blast disease	F_2_	–	20, 20	Takagi *et al*. (2015)
Rice	Cold tolerance	F_3_	10 800	430, 385	Yang *et al*. (2013)
Rice	Pale green leaves and semidwarfism	F_2_	–	20, 20	Abe *et al*. (2012)
Cotton	Nulliplex‐branch	F_2_	168	30, 30	Chen *et al*. (2014)
Cotton	Short‐fibre mutant	F_2_	536	100, 100	Thyssen *et al*. (2012)
Cucumber	Early flowering	F_2_	–	10, 10	Lu *et al*. (2008)
Maize	Multiple traits	Natural population	7000	200, 200	Yang *et al*. (2015)
RNA‐seq based analysis					
Maize	*gl3* gene	F_2_	–	32, 31	Liu *et al*. (2013)
Wheat	Grain protein content	RSLs	–	14, 14	Trick *et al*. (2012)
Wheat	Yellow rust	F_2_	232	–	Ramirez‐Gonzalez *et al*. (2012)
Sunflower	Downy mildew	F_2_	2141	16, 16	Livaja *et al*. (2005)
Sand pear	Pericarp russet pigmentation	F_2_	–	10, 10	Wang *et al*. (2011)
Onion	Restorer‐of‐fertility	F_2:5_	251	10, 10	Kim *et al*. (2011)
Radish	Cytoplasmic male sterility	F_2_	224	10, 10	Lee *et al*. (2010)

RILs‐Recombinant inbred lines;? RSLs‐Recombinant substitution line.

‘–’: The information unavailable.

With the development of next‐generation sequencing (NGS) technologies, BSA has been used for quick discovery of associated markers and candidate genes by sequencing the parents and bulks of phenotypic extreme individuals from the segregating populations, through BSA based on DNA‐ and RNA‐seq. As a typical example for NGS‐assisted BSA method, 430 extreme sensitive and 385 extreme tolerant rice seedlings to low temperature were selected from a very large F_3_ population with 10 800 individuals, and genotyped with about 450 000 SNPs, with six QTL for low temperature, four QTL for partial resistance to the fungal rice blast disease and two QTL for seedling vigour identified (Yang *et al*., 2013). Using 946 F_2_ plants, rice male sterility gene *ms*‐IR36 was mapped to a 33‐kb region on the short arm of chromosome 2 that includes 10 candidate genes (Frouin *et al*., 2009). Meanwhile, QTL‐seq has been used to identify an early flowering QTL located near *Flowering Locus T* in cucumber (Lu *et al*., 2008). DNA‐seq BSA has also been used to study nulliplex‐branch and short‐fibre mutant in cotton (Chen *et al*., 2014; Thyssen *et al*., 2012). In few cases, DNA‐seq BSA has been used to identify several genes simultaneously from single population (Takagi *et al*., 2013b; Yang *et al*., 2013) and using multiple parallel bulks to identify the same gene (Ghazvini *et al*., 1991; Hiebert *et al*., 2007).

With NGS and *de novo* transcriptome assembly, a similar pipeline as DNA‐seq BSA can be developed to enrich molecular markers and identify eQTL and candidate genes through RNA‐seq BSA. In maize, this method was used to rapidly and efficiently map genes for mutant phenotypes, in which 32 mutants (*gl3‐ref/gl3‐ref*) and 31 nonmutant siblings (*gl3‐ref/Gl3‐B73* or *Gl3‐B73/Gl3‐B73*) were selected with the *gl3* locus mapped (Liu *et al*., 2013). Other examples include genetic mapping of grain protein content (Trick *et al*., 2012) and major disease resistance for wheat yellow rust (Ramirez‐Gonzalez *et al*., 2012) in wheat, downy mildew disease resistance in sunflower (Livaja *et al*., 2005), pericarp russet pigmentation in sand pear (Wang *et al*., 2011) and cytoplasmic male sterility in radish (Lee *et al*., 2010).

### Genomics

#### Functional analysis

Whole‐genome information and high‐throughput tools have contributed to the development of functional genomics, including transcriptomics (Zimmerli and Somerville, 2005) and proteomics (Roberts, 2002).

Protein arrays have five major applications in human and animals, including diagnostics, proteomics, protein functional analysis, antibody characterization and treatment development. For example, BSA‐based functional genomics analysis identified 193 genes showing greater mRNA abundance in adult oocytes and 223 genes showing greater mRNA abundance in prepubertal oocytes (Patel *et al*., 2000).

In *Lactobacillus rhamnosus*, 100 strains isolated from diverse sources were used to understand the genetic complexity and ecological versatility of the species through genomic and phenotypic analysis (Douillard *et al*., 2011). By mapping their genomes onto the *L. rhamnosus* GG reference genome, a wide range of metabolic, antagonistic, signalling and functional properties were characterized.

In plants, protein functional analysis is one of the major genomics applications. It has been used to identify protein–protein interactions (e.g. identification of members of a protein complex) (Kushwaha *et al*., 1989, 2013), protein–phospholipid interactions (Conde and Patino, 2011), small molecule targets (Kaschani *et al*., 2008), enzymatic substrates (particularly the substrates of kinases) (Wijekoon and Facchini, 2002) and receptor ligands (Lee *et al*., 2003).

With the rapid development in liquid chromatography coupling with tandem MS (LC‐MS/MS), large‐scale proteome identification and quantification can be achieved (Cooper *et al*., 2008; Nilsson *et al*., 2011; Ning *et al*., 2007). In plants, proteins with potential agronomic values have been detected by high‐throughput protein sequencing. Around 200 gliadins and glutenins have been identified in wheat flour using MS, and their abundance was also examined (Dupont *et al*., 1987). Approximately 4975 nuclear proteins were detected in soybean leaves, and protein differently expressed in between soybean rust susceptible and resistant plants might be involved in disease resistance (Cooper *et al*., 2008). Using two‐dimensional difference gel electrophoresis coupled with LC‐MS/MS, 42 brassinosteroid (BR)‐regulated proteins were identified in *Arabidopsis*. These proteins are predicted to play potential roles in specific cellular processes, including signalling, cytoskeleton rearrangement, vesicle trafficking and biosynthesis of hormones and vitamins (Deng *et al*., 2013).

#### Genotype by environment interactions

Phenotypic expression is environment‐dependent. The environments can be defined as the sum total of circumstances surrounding or affecting an organism or a group of organisms. Cultivars as pure‐breeding genotypes, when grown under a wide range of environments, are exposed to different soil types, fertility levels, moisture contents, temperatures, photoperiods, biotic and abiotic stresses and agronomic practices. As gene expression may be modified, enhanced, silenced or timed by regulatory mechanisms in the cell to respond to internal and external factors, the genotypes (cultivars) may specify a range of phenotypic expressions that are called the norm of reaction, or plasticity, which is simply the expression of variability (Bradshaw, 1965).

Genotype by environment interactions have been investigated with focus upon individual phenotypic traits. Generalized principles behind GEI could be revealed through high‐throughput techniques that have greatly expanded the depth so that traits of agronomic importance can be analysed in terms of genotypic and environmental effects (Xu, 2016). Three genetic mapping approaches, linkage mapping using bi‐parental populations, GWAS with natural populations and integrated linkage‐GWAS, for example, can be used along with DNA‐, RNA‐ and protein‐seq. Combining a GWAS with transcriptional networks and metabolite or protein composition phenotypes will facilitate rapid identification and validation of many genes that are potential causal genetic candidates (Chan *et al*., 2015; Fu *et al*., 2013; Joosen *et al*., 2010). Therefore, combining ecophysiological modelling with genetic mapping to create QTL‐based ecophysiological models could help narrow down genotype–phenotype or gene–phenotype gaps. For example, RNA‐seq has been used to simultaneously examine tens of thousands of measurements in the form of gene expression levels. Gene expression can be monitored through whole‐genome sequencing across individuals, developmental stages and environments to identify responsive genotypes. By incorporation of additional genes and regulatory links, gene regulatory network may have enriched more ancient and, consequently, more connected gene components for GEI (Grishkevich and Yanai, 2014).

The whole‐genome strategies and associated methods hold great power in GEI analysis. Identification of GEI across four dimensions (genome × environment × space × time) will be able to query how gene expression at different developmental stages across different spaces and environments respond to GEI (Xu, 2016). To dissect GEI into its individual genetic components, the genetic complexity of the phenotypic responses to the environment should be examined with underlying genes and their allelic composition and combinations (haplotypes). As a consequence, GEI will be identified to correspond to (often several) QTL with environment‐specific effects (El‐Soda *et al*., 2012).

Understanding GEI can be facilitated by experiments under well‐managed environments (Xu 2015, 2016). However, the results obtained in a managed environment such as in climate chamber or greenhouse should not be generalized to the field environments because of the expected large GEI (Araus and Cairns, 2014; Tuberosa, 2002). Such warning is supported by several reports on *Arabidopsis*, where a poor correlation has been observed between variation for flowing time (FT) scored in field experiments and FT variation observed under greenhouse conditions (Brachi *et al*., 2010; Hancock *et al*., 2008a; Méndez‐Vigo *et al*., 2011).

### Crop improvement

#### Development of diagnostic and constitutive markers for breeding

The combination of advanced sequencing technology with BSA provides a powerful tool for rapid identification of genes or causal mutations, which can be used to develop markers for breeding, as using traditional bulked segregant analysis discussed by Xu and Bai (2015). Compared with entire population analysis, BSA provides a short cut to identifying and developing markers for important agronomic traits, which basically follows the approaches currently available. In addition to most frequently used DNA‐based markers, results from RNA and protein analyses can be also used to develop markers. For more effective marker‐assisted selection (MAS), markers should be developed from genic or functional regions and associated with gene functions. To reveal allelic variation within a gene, multiple markers shall be developed to construct haplotypes that represent different combinations of marker alleles. The higher the density of markers can be established in a specific region, the more meaningful haplotypes can be constructed.

#### Agronomic genomics

With the development of RNA‐seq, effects of agronomic practices on plant growth and development can be determined by examining the change in gene expression under different agronomic practices such as fertilization, irrigation, weeding and pest control. The term agronomic genomics can be used to represent the genomic study of the effects of agronomic practice on gene expression for the target traits, by which high‐efficient, cost‐effective and environment‐friendly agronomic practices can be developed to optimize the gene expression and thus the crop production (Xu, 2010). The drivers of gene expression patterns can be determined more straightforward in the complex fluctuating environments where organisms typically live. However, one of the major difficulties in analysing data associated with agronomic practice is the complex, noisy and multiple environmental factors that affect the transcriptome change simultaneously in the field. Agronomic genomics can facilitate breeding crops that respond better to agronomic practices.

As an example in agronomic genomics, transcriptome data collected from the leaves of rice plants in a paddy field, along with the corresponding meteorological data, were used to characterize the changes in transcriptome under natural field conditions and to develop statistical models for the endogenous and external influences on gene expression (Nagano *et al*., 2010). The model was built using 461 microarray data with distinct sampling time points and the corresponding meteorological data including wind speed, air temperature, precipitation, global solar radiation, relative humidity and atmospheric pressure. The predictive performance of the model was evaluated using 108 and 16 microarray data collected from plants grown in the field in two crop seasons, compared with the microarray data collected from plants grown in light‐ and temperature‐controlled growth chambers. Endogenous diurnal rhythms, ambient temperature, plant age and solar radiation predominantly control the transcriptome dynamics, allowing prediction of the influence of changing environments and also the relevant biological changes.

#### Marker‐assisted selection and selective phenotyping

For more effective MAS, a large population can be classified into several subpopulations based on marker–trait associations or population properties. BSA can be then used to improve the efficiency of selection for each subpopulation. Furthermore, selective genotyping can be applied to each subpopulation to identify desirable individuals.

Selective phenotyping, that is phenotyping only a part of individuals from the target population, is most effective when prior knowledge of genetic architecture allows focus on specific genetic regions (Jannink, 2012; Jin *et al*., 2013) and specific allele combinations or haplotypes, particularly for the traits that are difficult or expensive to evaluate. As genotyping or sequencing becomes much cheaper, it may be more efficient to first genotype the whole population to identify the most informative subset of individuals, and then phenotype this subset precisely. Compared with unselective phenotyping, selective analysis should have dramatically improved power for the same number of individuals phenotyped, as a much wide genetic variation would have been sampled for genotyping. Selective phenotyping becomes much simplified when phenotypic extremes can be easily identified by a simple phenotypic screening, for example abiotic stress tolerance, where a large number of plants/families can be eliminated easily under severe stress (Xu and Crouch, 2008).

High‐density planting and selection at early stages of plant development, which allows one to work with more plants/families at the same cost, should also be investigated as a viable option for some traits (Xu and Crouch, 2008). Where the target trait is largely influenced by planting density or strong selection pressure, this type of selective phenotyping will clearly confound the capacity to make genetic gain. However, many major gene‐controlled traits can be selected in this way without much disturbance.

## Perspectives

Bulked sample analysis has both the advantages, as discussed in previous sections, and some limitations. Although BSA has been widely used with many examples available in genetics and genomics, it has been largely focused on relatively simple traits controlled by major genes. For quantitative traits that are controlled by genes with different effects, selection of bulked samples can be improved through precision phenotyping assisted by envirotyping and performed with controlled environments or well‐managed experimental trials (Xu, 2010), by which genes with relatively large effects could be targeted. For complex traits that involve many genes each with minor effect and affected significantly by environments, BSA may not be effective as the entire population analysis, as selection of the extremes containing all favourable alleles from a large number of loci would be impossible (Farkhari *et al*., 2012). Compared with traditional methods in genetic analysis, BSA requires to identify only individuals showing contrasting extreme phenotypes in the target populations, while not taking into account the accurate trait values for the rest (Yang *et al*., 2013). As a consequence, genetic analysis by BSA might upward or downward the real value such as phenotypic variance, LOD score and additive effects (Vikram *et al*., 2009). Furthermore, BSA consistently fails to identify epistatic interactions (Schneeberger, 2009), and it is more insensitive to occasional phenotyping mistakes (Schneeberger *et al*., 1990). However, BSA can be improved by increased population and tail sizes and marker density (Sun *et al*., 2013a), by using multiple bulked samples, and through precision phenotyping and envirotyping. In addition, GWAS using individual genotypes may be also used for validation of BSA results and elucidation of complicated scenarios more reliably.

With the significant reduction in cost in genome sequencing, especially the development of RNA‐seq, genotyping has been more simplified than before. As a result, BSA can be performed by genotyping multiple bulks from a large‐size population, by which the power of detection can be improved (Ghazvini *et al*., 1991; Hiebert *et al*., 2007; Sun *et al*., 2013a) as rare events or alleles of interest can be verified among multiple bulks or by individual genotyping after appropriate sub‐bulks are identified. When it comes to optimal designs of NGS‐assisted BSA, it should take into account the number of bulks, number of individuals in each bulk and sequencing depth (Kim *et al*., 2015).

Compared with DNA‐microarrays, developing protein microarrays requires a lot more steps in its creation and faces many challenges (Cretich *et al*., 2008; Gahoi *et al*., 2007; Hall *et al*., 2011; Liotta *et al*., 2012; Lueking *et al*., 2011; Zhu and Snyder, 2003). Technical challenge is to find a surface and a method of attachment that allows the proteins to maintain their secondary or tertiary structure, and to produce an array with a long shelf life so that the proteins on the chip do not denature over a short time. Experimental challenges are to acquire antibodies or other capture molecules against every protein in the target genome, quantify the levels of bound protein, extract the detected protein from the chip for further analysis and reduce nonspecific binding by the capture agents (https://en.wikipedia.org/wiki/Protein_microarray). By the end, the capacity of the chip should be significantly increased to allow the whole proteome to be represented and less abundant proteins to be detected.

Technically, microarray‐based high‐throughput analysis of protein–protein and other biomolecular interactions holds immense potential for multiplex interactome mapping and also great opportunity for an inclusive representation of the signal transduction pathways and networks (Gahoi *et al*., 2007). Equipped with multiplexing, quantitative proteomics comes to the era of ‘ultra’ high‐throughput, making it possible to comprehensively compare all major tissues/organs of humans (Paulo *et al*., 2012) and plants as well in a day.

Although BSA has been widely used at DNA and RNA levels, its use at protein level has not been reported. Since many MS‐based protein quantification tools are designed for comparison between two samples, we would expect that many of them are suitable for protein‐based BSA. A major challenge in protein‐based BSA would be that enrichment in cellulose and many plants’ secondary metabolites such as polyphenols, lipids, organic acids, terpenes or pigments makes it hard to extract pure proteins from tissues.

Traditional genotyping methods, such as individual marker‐based or marker chip‐based, should be complemented by GBS, including both regular whole‐genome sequencing and the simplified genome sequencing with target enrichment or reduction in genome complexity (De Donato *et al*., 2008; Elshire *et al*., 2014; He *et al*., 1997; Poland and Rife, 2007). GBS technology is flexible and efficient, providing acceptable marker density for genomic selection or GWAS at roughly one‐third of the genotyping cost of currently available technologies (De Donato *et al*., 2008), and the cost becomes less when samples are multiplexed (Zhang *et al*., 2014b). GBS can be used in GWAS, genomic diversity study, genetic linkage analysis, molecular marker discovery and genomic selection under a large scale of plant breeding programs (He *et al*., 1997). When the number of individuals contained in the two bulks is large enough, for example, more than 500, BSA can be combined with GBS technologies and used for GWAS (Duncan *et al*., 2011; Schlötterer *et al*., 2014).

It can be expected that BSA, which have been widely used with mixed success in genetic mapping and gene identification, will become increasingly important in genetics, genomics and crop improvement and will replace the analysis of all individuals (entire population) in many cases. BSA by sequencing (BSA‐seq) will become more attractive with the development of dedicated software for BSA‐seq data analysis, novel techniques for analysis of low‐frequent or rare variants, new approaches to accurate estimates of haplotypes, and new sequencing technologies allowing generation of longer sequencing reads to facilitate the reconstruction of haplotype information (Schlötterer *et al*., 2014). As genomewide selective genotyping and BSA become possible, an effective information management and data analysis system will be required to make full use of BSA in genetics, genomics and plant breeding.

## Supporting information


**Figure S1** Evolution of genetic markers and marker analysis.

**Appendix S1** Populations in genetics, genomics and crop improvement.
