# Supplementary figures and images for: Bulked sample analysis in genetics, genomics and crop improvement
Source: Plant Biotechnol J. 2016 Apr 28;14(10):1941–55. doi: 10.1111/pbi.12559 (PMC5043468; doi:10.1111/pbi.12559)

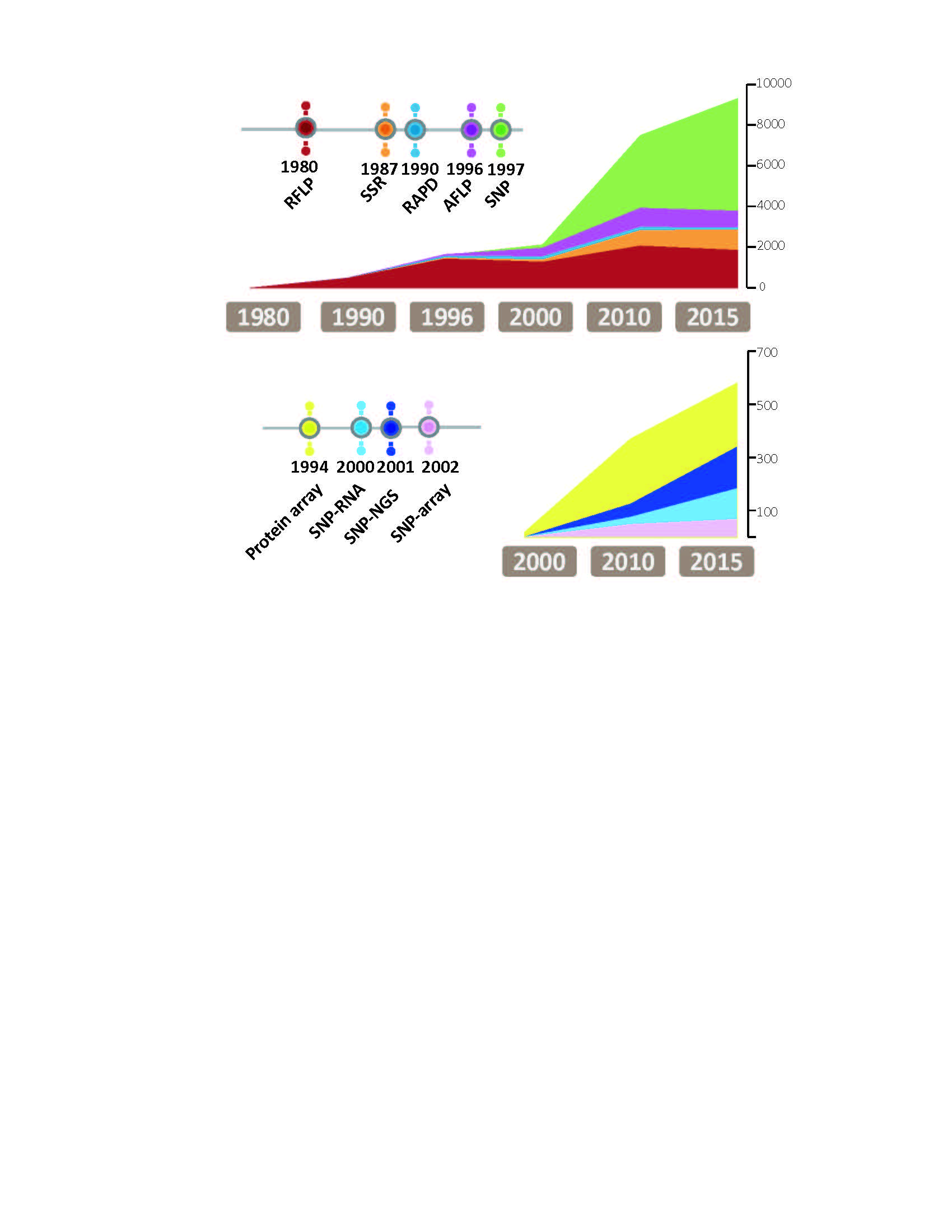

Supplement: Supplementary file 1 — Figure S1 Evolution of genetic markers and marker analysis. [file PBI-14-1941-s001.jpg]
