# Supplementary material for: Bulked sample analysis in genetics, genomics and crop improvement
Source: Plant Biotechnol J. 2016 Apr 28;14(10):1941–55. doi: 10.1111/pbi.12559 (PMC5043468; doi:10.1111/pbi.12559)
Supplement: Supplementary file 2 — Appendix S1 Populations in genetics, genomics and crop improvement. [file PBI-14-1941-s002.docx]

**Bulked sample analysis in genetics, genomics and crop improvement**

Cheng Zou^1^, Pingxi Wang^1^, Yunbi Xu^1, 2^*

***Supplemental Information:***

**Populations in genetics, genomics and crop improvement**

**Biparental populations**

- F_2_: it is a most informative population type, but phenotypes cannot be distinguished individually due to the presence of heterozygotes and single-plant caused errors, and thus its phenotyping is usually done with F_2_-derived F_3_ families (F_2:3_).
- BC_1_: for each target locus, it consists of two genotypes which can be distinguished theoretically, but the genetic composition will change with generation advancement. Phenotyping is usually done based on its derived families.
- RIL: it consists of stable genotypes and its individuals can be distinguished as two types of homozygotes at each marker locus, but its development is time-consuming.
- DH: it consists of high-purity genotypes and represents the gene segregation and recombination rate of F_1_, but its development depends on the properties of crop species and appropriate DH approaches which might be very difficult or not available for some crop species.

**Multiparental population**s

There two three types of multi-parental populations, the population developed from a composite cross with three or more parental lines, nested association mapping (NAM) population (Yu et al. 2008), and multi-parent advanced generation inter-cross (MAGIC) population (Kover et al. 2009). NAM is developed by crossing a common line with a diverse panel of lines and a set of RIL populations is then developed. Different sets of RIL populations share a common parent, while RILs within each population are derived from two parents. The NAM population combines the advantages of both biparental and natural populations, which can be used for joint linkage - linkage disequilibrium (association) mapping. The first NAM population in plants is developed by using 25 crosses between diverse inbred lines and a common inbred B73, which resulted in 25 RIL populations with 5000 lines in total (Yu et al. 2008). It has been successfully applied to the study of many traits, including flowering time (Buckler et al. 2009) and resistance to Southern corn leaf blight (Kump et al. 2011).

The MAGIC population starts with multiple biparental crosses, and then two biparental F_1_s are intermated with each other to produce double hybrids. The two double hybrid F_1_s are intermated again to form a hybrid with eight parents involved. This process continues to have as many as possible parental lines included in a composite hybrid. Finally a set of RILs or DHs are developed from the composite hybrid by continuous selfing or a doubled haploid approach. The first MAGIC population has been developed by using a set of 527 RILs descended from a heterogeneous stock of 19 intermated accessions of *Arabidopsis thaliana*, and used to fine map QTL (Kover et al. 2009).

**Special mating-design populations**

They are populations derived from special mating designs, such as, diallel (all possible biparental F_1_s among multiple parents), NCD I (two inbred lines are crossed to produce an F_2_, and then some individuals are randomly selected from the F_2_ population as males to intermate with other randomly selected females), NCD II (n parental lines are divided into two groups, one group as males and the other as females, to produce all possible hybrids), NCD III (n individuals are selected from an F_2_ population to backcross with their parents, P_1_ and P_2_), triple testcrosses (TTC; an extension of NCD III, n individuals (n > 20) are selected from an F_2_ population to backcross with their parents and F_1_ ), and simplified triple testcrosses (sTTC; n cultivars or strains are selected from the germplasm pool to cross with two cultivars or strains, P_H_ and P_L_, which show extreme phenotypes (with the highest and lowest phenotypic values, respectively).

**Natural populations**

They are natural (produced individually with no intention of combining use), including a panel of inbreds, varieties, landraces, or wild relatives, with significant variation for the target trait but random variation for other traits.

**Testcross populations**

They are produced by testcrossing all the individuals from any of populations described above with their parents or selected genotypes/varieties, which can be used for genetic analysis of hybrid traits, hybrid performance, and combining ability, and for comparative genetic analysis with their parental lines and populations.
